# Occurrence of Methicillin Resistant and Enterotoxigenic *Staphylococcus aureus* in Traditional Cheeses in the North West of Iran

**DOI:** 10.1155/2014/129580

**Published:** 2014-02-13

**Authors:** Dariush Shanehbandi, Behzad Baradaran, Saeed Sadigh-Eteghad, Habib Zarredar

**Affiliations:** ^1^Immunology Research Center, Tabriz University of Medical Sciences, Tabriz, Iran; ^2^Neurosciences Research Center (NSRC), Tabriz University of Medical Sciences, Tabriz, Iran

## Abstract

Traditional dairy products are potential sources of a variety of microorganisms which participate in food poisoning. *Staphylococcus aureus* is a conspicuous example of toxigenic bacteria causative for food-borne diseases. Moreover, resistance to methicillin is a prominent index in food hygiene studies. In the present study, we have aimed at characterization and identification of enterotoxigenic methicillin resistant *S. aureus *(MRSA) isolated from traditional cheeses in Azerbaijan region in the northwest of Iran during 2012. A number of phenotypical and molecular assays were utilized for screening of *S. aureus*. Subsequently, the prevalence of the genes responsible for the five staphylococcal enterotoxins (SEA-SEE) and also methicillin resistance gene was assessed. The outcomes of phenotypical methods were in conformity with those of the molecular procedures. The results indicated that 16% of cheese samples were contaminated by *S. aureus*. 110 isolates were authenticated by both phenotypical and molecular methods. All of the mentioned isolates were positive for *coa, nuc*, and 16S rDNA primers. 21% of these isolates were *mecA* positive and 60.8% of these MRSA were positive for SEs. Regarding the frequent outbreaks of enterotoxigenic MRSA, new hygiene policies and management practices should be considered to increase food safety and avoid extra treatment costs.

## 1. Introduction

Food-borne diseases are a public health problem worldwide. To date, approximately 250 different food-borne diseases have been described while bacteria were responsible for the two-thirds of them. Among these bacteria, *Staphylococcus aureus* is one of the most common agents in food poisoning outbreaks [1, 2]. *S. aureus* is a Gram and coagulase positive cocci, which belongs to the Staphylococcaceae family. This bacterium is a major causative pathogen of clinical or subclinical mastitis of dairy milk and milk products [3, 4]. There are frequent reports of prevalence of *S. aureus* in extensively used traditional cheeses [5, 6]. Staphylococcal Enterotoxins (SEs), the main causatives for food poisoning, are a group of single chain, low molecular mass proteins and are produced during all phases of growth [7]. Based on serological classification, to date several SEs have been recognized such as SEA, SEB, SEC, SED, and SEE [8]. SEs are highly thermostable and resistant to most proteolytic enzymes and different environmental conditions. The detection of SEs is known as a reliable means for the confirmation of staphylococcal outbreaks and determination of the enterotoxigenicity of strains [9]. On the other hand, due to the intensive use of antibiotics in public health and animal breeding, antibiotic resistance in pathogens including the genus *Staphylococcus* has become an increasing medical problem during the last decades [10, 11]. Moreover, the methicillin resistant *S. aureus* (MRSA) should be also considered resistant to all penicillins, cephalosporins, cephems, and other *β*-lactams [12]. Therefore, preventing the prevalence of resistant strains seems to be of a central importance. Accordingly, a variety of sufficient techniques should be employed for surveying the presence of MRSA and related toxins in foodstuff. Nowadays, molecular biology techniques are considered as important tools in the microbiological studies [9]. Traditional methods such as immunological procedures require detectable amounts of toxins. In addition, the detection of enterotoxins by the mentioned methods is generally more complicated and time consuming than the molecular methods and sometimes they are not practical for detection and identification of a large group of SEs [13]. In the present study both phenotypical and molecular procedures were employed for assessment of enterotoxigenicity and methicillin resistance of *S. aureus* in traditional cheeses in the East Azerbaijan, Iran.

## 2. Materials and Methods

### 2.1. Samples Collection and Culture

Traditional cheeses in Azerbaijan region are generally produced from goat or sheep's fatty milk with a temperature of 30°C by adding commercial rennet [14]. In the present study, one hundred samples of 20 grams were collected from local producers in the rural areas of East Azerbaijan from January through December 2012. For the primary culture, 50 *μ*L of homogenized samples was surface-plated on nutrient agar and incubated at 37°C for 24–48 h under aerobic conditions. Colonies suspected as *S. aureus* were selected and transferred into individual tubes of nutrient broth. Colonies were sub-cultured on 5% sheep blood agar and mannitol salt agar (Himedia, India). Culture characteristics, Gram staining, and catalase and coagulase test utilizing fresh rabbit plasma were the criteria used for the identification of presumptive isolates. The carbohydrates differential fermentation was also used for confirmation of all coagulase positive (CP) isolates [15]. *S. aureus* ATCC 25923 and *Escherichia coli* ATCC 35218 served as reference strains for biochemical tests.

### 2.2. Methicillin Resistance Test

Resistance of staphylococcal isolates to methicillin was tested by disk diffusion assay according to the guidelines of the National Committee for Clinical Laboratory Standards (NCCLS, 2003) using Mueller Hinton agar [11].

### 2.3. DNA Extraction

A modified one-step protocol was utilized for genomic DNA extraction from overnight cultures of putative staphylococcal isolates [16]. Bacterial pellets in Eppendorf tubes were alternatively placed in liquid nitrogen and 60°C water bath for five to seven times. Then, 900 *μ*L of CTAB lysis buffer (Applichem, A4150) was added and the microtubes were incubated in 65°C water bath for 45 min. 500 *μ*L of phenol-chloroform-isoamyl alcohol in a ratio of 25 : 24 : 1 was added to the samples and the procedure was followed by 2-propanol precipitation. RNase-A (DNase-free) was added into tubes in final concentration of 20 *μ*g/mL. The extracted DNA was evaluated by 0.8% agarose gel electrophoresis.

### 2.4. Primer Design for 16S Ribosomal RNA Gene

Several nucleotide sequences corresponding to staphylococcal 16S rDNA gene were acquired from NCBI database. ClustalX (ver. 1.81) and Mega (ver. 4) software were used for alignment of the mentioned sequences. Subsequently the Oligo5 software was utilized for designing of *Staphylococcus* 16S rDNA primers. The specifications of the mentioned primers, namely, ST16F and ST16R, are shown in Table 1. The mentioned primers can be used for identification of a wide range of staphylococci in genus level.

### 2.5. PCR Amplification of 16S rDNA Gene

The designated primers were used for amplification of an approximately 1412 bp of 16S ribosomal RNA gene. PCR was carried out in a final volume of 50 *μ*L in a Techne (TC-412) thermal cycler. Each reaction contained 40 ng of DNA, 0.5 *μ*M of ST16 primers, and 25 *μ*L of 2X Amplicon Master Mix. PCR program consisted of an initial denaturation step at 94°C for 4 min which was followed by 35 cycles of denaturation at 94°C for 1 min, annealing at 55°C for 45 s, extension at 72°C for 80 s, and eventually a final extension step of 72°C for 10 min.

### 2.6. Multiplex-PCR for Staphylococcal Enterotoxins (A–E)

Multiplex PCR was carried out in a final volume of 50 *μ*L. Each reaction contained 50 ng of DNA, 0.5 *μ*M of A2, B2, C2, D2, and E2 primers, 0.7 *μ*M of U1 primer, and 0.9 *μ*M of U2 primer (Table 1). 2X Amplicon Master Mix was used in the same quantity described for 16S ribosomal RNA gene. Initial denaturation was 3 min at 94°C, which was followed by 35 cycles of denaturation at 94°C for 50 s, annealing at 52°C for 55 s, and extension at 72°C for 80 s with a final extension step of 72°C for 10 min.

### 2.7. PCR Amplification of Coagulase, Nuclease, and Methicillin Resistance Genes

PCR for amplification of nuclease (*nuc*), coagulase (*coa*), and methicillin resistance (*mecA*) genes was performed in separate reactions. Primer sequences are indicated in Table 1. All PCR products were analyzed by 1% (w/v) agarose gel electrophoresis in TAE buffer stained with ethidium bromide.

## 3. Results

**Figure 1:**
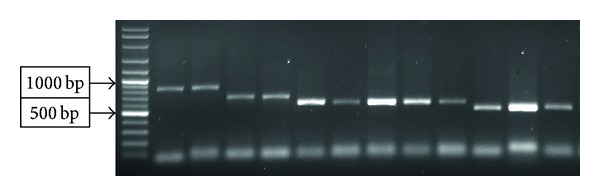
Examples of the four sizes of *coa* gene from representative *S. aureus* isolates.

120 susceptible colonies were isolated from cheese samples and subjected to phenotypical and molecular authentication tests. Making use of biochemical assays, 110 colonies were found to be positive for coagulase. PCR for amplification of the entire 3′ repeat elements of *coa* gene resulted in one of 875, 660, 603, or 547 bp amplicons for all biochemically *coa* positive isolates (Figure 1). PCR with 16S-rDNA primers resulted in approximately 1412 bp amplicons for all 110 isolates. Evaluation of strains for *nuc* gene also revealed that all of the putative samples were positive for this trait. Eventually, 16 out of the 100 cheese samples (16%) examined were found to be contaminated with *S. aureus*.

Methicillin susceptibility test also indicated that 21% of *S. aureus* isolates (23 out of 110 isolates) were resistant to this antibiotic. Application of molecular methods in the present study showed that all of the MRSA phenotypes were positive for presence of *mecA* gene which is an important index for antibiotic resistance.

Furthermore, the multiplex PCR for SEs revealed that 14 isolates of these 23 methicillin resistant strains were positive for SEs. In this case eight (34.8%) of the isolates were positive for SEA, three (13%) were positive for the SEB gene, one (4.3%) was found to be SEC positive, none of samples were positive for SED, and two isolates (8.7%) contained the gene responsible for SEE.

## 4. Discussion

Methicillin resistant *S. aureus* strains, because of their high mortality, have become a major concern worldwide [17]. MRSA, including animal associated strains, have been frequently detected in dairy products such as raw milk or traditional cheeses [18–21]. Since the microbes of dairy products such as cheese are not being eliminated by heating or processing, they may serve as a vehicle to disperse MRSA [22].

Application of conventional criteria such as cultural and biochemical features for identification of *S. aureus* and MRSA isolates is slow and time consuming. In contrast, molecular methods have been proven as rapid and reliable means in the study of outbreaks and epidemiology of a wide range of microorganisms.

In the present study, 16S rDNA gene amplification was employed for characterization of the isolates. The deigned primers could be utilized for direct and rapid identification of a wide range of *Staphylococcus* isolates at genus level. Consequently, all susceptible strains were positive for 16S rDNA primers. Moreover, PCR on *nuc* gene for rapidly diagnosis of *S. aureus* [23] confirmed all presumptive strains. Molecular analysis of *coa* gene resulted in four sizes of products. The presence of different amplicon sizes is due to repetitions of 3′ elements of *coa* gene in various strains [24]. In a similar study performed in the north west of Iran, the four sizes were detectable [25]. The prevalence of *S. aureus* was about 16% in the present study. However, the occurrence of this microorganism in dairy products or other foodstuff is different among studies [24, 26–34]. Several factors such as source of sampling, geographical origin, sensitivity of the identification methods, and the quantity of samples can affect the outcomes. For instance, in one study *S. aureus* was found in 7.3% of commercial milk samples in Brazil [35]. In other study in Italy, 13.3% of fresh cheeses were contaminated with *S. aureus* [36]. The contamination degree also varies among different kinds of dairy products, while cream, cheese, and milk are arranged in a descending order from the viewpoint of contamination. It is deduced that further handling and manipulating of milk products could lead to more *S. aureus* contamination [37].

In analysis of methicillin resistance, molecular methods are also proved to be significantly useful. In one study, PCR based *mecA* gene amplification confirmed more than 99% of MRSA isolates [26]. In our study, *mecA* gene was detected in 21% of the *S. aureus* isolates (23 out of 110). In a similar study by Türkyılmaz, among 93 *S. aureus* strains isolated from bovine milk with mastitis in Aydin, Turkey, 16 were resistant to methicillin [12]. The high prevalence of resistance genes should be considered as a potential health risk for humans and livestock. Consequently, necessary precautions should be taken by governments and individuals to prevent the further spread of MRSA. Hygiene promotion and avoiding the unsupervised use of antibiotics seem to be elementary steps in this regard.

On the other hand, the investigation of the strains for enterotoxins is an important experiment in pathogenesis studies [38]. In our study, 60.8% of MRSA were positive for the presence of SEs.

In the study performed by Løvseth et al. in Brazil [35], strains isolated from 14.3% samples were enterotoxigenic. In another study, which included SEA and SEB genes, the PCR results proved that 15.6% of the *S. aureus* isolates possessed the SEA gene and 9.3% the SEB gene [37]. In brief, detecting the SE genes by molecular techniques could help understand the virulence mechanisms and pathogenic potential of this microorganism.

## 5. Conclusion

The data presented in this study represents the information about prevalence of methicillin resistant and enterotoxigenic *Staphylococcus aureus* isolates from traditional cheeses in the north west of Iran and highlights a strong need for epidemiological and pathogenic studies to monitor distribution, infectious species, and infection kinetics. This information should provide awareness and persuade the authorities to set new hygiene policies. Furthermore, it could be helpful in disease management practices and reducing the overall costs of medication. It also shows that molecular methods provide rapid, accurate, and economic means for *S. aureus* screening in food safety evaluations.

## Figures and Tables

**Table 1:** List of primers used in the present study.

Gene	Primer	Primer sequence (5′ to 3′)	Size (bp)	Reference
Coagulase	*coa*-F	ATAGAGATGCTGGTACAGG	674–917	[15]
	*coa*-R	GCTTCCGATTGTTCGATGC		
Methicillin resistance	*mecA*-F	AAAATCGATGGTAAAGGTTGGC	533	[39]
	*mecA*-R	AGTTCTGCAGTACCGGATTTGC		
16S rDNA	ST16-F	TTG CTT CTC TGA TGT TAG CG	1412	Designed
	ST16-R	AAT CAT TTG TCC CAC CTT C		
Nuclease	*nuc*-F	GCGATTGATGGTGATACGGTT	276	[32]
	*nuc*-R	AGCCAAGCCTTGACGAACTAAAGC		
Enterotoxin A	SEA-A2	ATTAACCGAAGGTTCTGTAGA	582	[40]
	SEA-U2	TTGCGTAAAAAGTCTGAATT		
Enterotoxin B	SEB-B2	TTTTTCTTTGTCGTAAGATAA	732	
	SEB-U1	CCAACGTTTTAGCAGAGAAG		
Enterotoxin C	SEC-C2	TAAGTTCCCATTATCAAAGTG	403	
	SEC-U1	CCAACGTTTTAGCAGAGAAG		
Enterotoxin D	SED-D2	TAATGCTATATCTTATAGGG	251	
	SED-U2	TTGCGTAAAAAGTCTGAATT		
Enterotoxin E	SEE-E2	TAAACCAAATTTTCCGTG	474	
	SEE-U2	TTGCGTAAAAAGTCTGAATT		
